# Screening Performance of Diabetes Risk Scores Among Asians and Whites in Rural Kerala, India

**DOI:** 10.5888/pcd10.120131

**Published:** 2013-03-21

**Authors:** Thirunavukkarasu Sathish, Srinivasan Kannan, Sankara P. Sarma, Kavumpurathu Raman Thankappan

**Affiliations:** Author Affiliations: Srinivasan Kannan, Sankara P. Sarma, Kavumpurathu Raman Thankappan, Sree Chitra Tirunal Institute for Medical Sciences and Technology, Kerala, India.

## Abstract

We compared the screening performance of risk scores for Asians and whites for diabetes, dysglycemia, and metabolic syndrome. Our subjects were 451 people aged 15 to 64 years who participated in a cohort study from May 2003 through September 2010 in a rural area of the Thiruvananthapuram district of Kerala, India. All outcome measures showed overlap in the range of area under the receiver operating characteristic curves of Asian and white diabetes risk scores (DRSs). Asian and white DRSs performed similarly in rural India.

## Objective

Although mass screening for diabetes is not practical or recommended, selective screening through risk scores is feasible, convenient, and cost effective. Most diabetes risk scores (DRSs) have been developed and validated among whites (1–7); evidence on their screening performance in Asians is limited (8,9). We compared the screening performance of Asian and white DRSs for diabetes, dysglycemia, and metabolic syndrome in rural India.

## Methods

In 2003, a large-scale cross-sectional survey on risk factors for noncommunicable diseases was conducted among 7,449 people aged 15 to 64 years in urban, slum, and rural areas of the Thiruvananthapuram district of Kerala, India (10). From the rural sample of the survey (n = 2,510), 495 people were selected for biochemical analysis (fasting plasma glucose and serum lipids) through systematic random sampling. We followed these 495 people from May 2003 through September 2010. During the follow-up study in 2010, 452 people (91.3%) participated (11). We used the baseline data (2003 study data) of 451 people, excluding that of 1 pregnant woman, for the present analysis. We defined dysglycemia according to World Health Organization guidelines (12) as the presence of impaired fasting glucose (fasting plasma glucose 110–125 mg/dL and not on antidiabetes medication) or diabetes (fasting plasma glucose ≥126 mg/dL or on antidiabetes medication, or both). We defined metabolic syndrome according to International Diabetes Federation criteria (13) as the presence of 3 or more of the following: raised triglycerides (≥150 mg/dL or treatment of this lipid abnormality), reduced HDL cholesterol (<40 mg/dL in men and <50 mg/dL in women, or treatment of this lipid abnormality), raised blood pressure (systolic blood pressure ≥130 mm Hg or diastolic blood pressure ≥85 mm Hg or treatment of previously diagnosed hypertension), or raised fasting plasma glucose (≥100 mg/dL or previously diagnosed type 2 diabetes).

We chose 11 DRSs (1-7,14-17) that could be applied to our data set (Box). Variables in Asian DRSs were age, family history of diabetes, physical activity, body mass index, waist circumference, and blood pressure. Variables in white DRSs were age, sex, family history of diabetes, smoking history, history of elevated blood glucose (having been told by a health care professional that they had diabetes), history of hypertension, use of antihypertensive medication, daily consumption of fruits or vegetables, physical activity, body mass index, waist circumference, and blood pressure. We derived the area under the receiver operating characteristic curve (AROC) by plotting 1-specificity on the *x*-axis and sensitivity on the *y*-axis. We used the DeLong method (18) to compare the AROCs of DRSs. We used the range of AROCs of Asian and white DRSs to compare their screening performance. We used univariate logistic regression analysis to examine the association of individual variables of Asian and white DRSs with outcome measures. For the optimal cutoff (score value with maximum sensitivity and specificity) of DRSs, we computed high risk (proportion of people requiring confirmatory biochemical testing), sensitivity (true positives/true positives + false negatives), specificity (true negatives/true negatives + false positives), positive predictive value (true positives/true positives + false positives), and negative predictive value (true negatives/true negatives + false negatives). We used SPSS version 17.0 (SPSS Inc, Chicago, Illinois) to perform data analyses. The study was approved by the Institutional Ethics Committee of the Sree Chitra Tirunal Institute for Medical Sciences and Technology, Thiruvananthapuram, India. We obtained written informed consent from all study participants.

**Box. Asian and White Diabetes Risk Scores (DRSs) Applied to Data Set**

**Table:** 

**Asian DRSs**	
Mohan et al (IDRS) (14)	India
Ramachandran et al (15)	India
Chaturvedi et al (16)	India
Aekplakorn et al (17)	Thailand
**White DRSs**	
Herman et al (ADA questionnaire) (1)	United States
Bang et al (2)	United States
Chen et al (AUSDRISK) (3)	Australia
Lindström et al (FINDRISC) (4)	Finland
Glümer et al (Danish Risk Score) (5)	Denmark
Balkau et al (DESIR) (6)	France
Baan et al (Rotterdam Prediction Model 1) (7)	Netherlands

Abbreviations: IDRS, Indian Diabetes Risk Score; ADA, American Diabetes Association; AUSDRISK, Australian Type 2 Diabetes Risk Assessment Tool; FINDRISC, Finnish Diabetes Risk Score; DESIR, Data from the Epidemiological Study on the Insulin Resistance Syndrome.

## Results

The mean age of the study sample was 39.4 (standard deviation [SD], 14.1) years. Table 1 provides details of screening performance of diabetes risk scores. We found no significant differences in the AROCs for diabetes, dysglycemia, or metabolic syndrome among Asian DRSs. However, the Rotterdam Prediction Model 1 (7) had significantly lower AROCs than other white DRSs for all outcome measures. We found overlap in the range of AROCs of Asian and white DRSs (excluding Rotterdam Prediction Model 1) for all outcome measures (Table 2). In Asian DRSs, 5 of 6 variables were associated with diabetes and dysglycemia and 5 with metabolic syndrome. Of the 12 variables in white DRSs, 8 were associated with diabetes and dysglycemia and 10 with metabolic syndrome. All 6 variables of Asian DRSs were present in white DRSs, although they had different cutoff values for age, body mass index, and waist circumference.

**Table 1:** Screening Performance of Diabetes Risk Scores for Diabetes, Dysglycemia, and Metabolic Syndrome Among 451 Participants in Rural Kerala, India

Diabetes Risk Score	Optimal CutOff^a^	High Risk^b^, %	Sensitivity^c^, %	Specificity^d^, %	PPV^e^, %	NPV^f^, %	AROC^g^ (95% CI)
**Diabetes^h^**							
IDRS (14)	≥60	49.0	85.7	59.4	32.6	94.8	0.80 (0.76–0.85)
Ramachandran et al (15)	≥24	35.3	71.4	73.0	37.7	91.8	0.79 (0.75–0.84)
Chaturvedi et al (16)	≥22	29.9	65.5	78.2	40.7	90.8	0.78 (0.73–0.83)
Aekplakorn et al (17)	≥7	45.5	79.8	62.4	32.7	93.1	0.78 (0.72–0.83)
ADA questionnaire (1)	≥6	37.3	70.2	70.3	35.1	91.2	0.74 (0.69–0.80)
Bang et al (2)	≥3	25.5	59.5	82.3	43.5	89.9	0.76 (0.70–0.82)
AUSDRISK (3)	≥15	27.3	67.9	82.0	46.3	91.8	0.83 (0.78–0.88)
FINDRISC (4)	≥6	37.7	73.8	70.6	36.5	92.2	0.81 (0.75–0.86)
Danish Risk Score (5)	≥18	39.7	73.8	68.1	34.6	91.9	0.76 (0.71–0.82)
DESIR^i^ (6)	≥3	41.7	66.7	64.0	29.8	89.4	0.72 (0.66–0.78)
Rotterdam Prediction Model 1 (7)	≥6	12.2	28.6	91.6	43.6	84.8	0.58 (0.50–0.65)
**Dysglycemia^j^**							
IDRS (14)	≥60	49.0	83.1	63.1	44.3	91.3	0.80 (0.76–0.84)
Ramachandran et al (15)	≥23	40.4	74.6	71.8	48.4	88.8	0.80 (0.75–0.84)
Chaturvedi et al (16)	≥15	58.3	90.7	53.2	40.7	94.1	0.79 (0.75–0.84)
Aekplakorn et al (17)	≥7	45.5	77.1	65.8	44.4	89.0	0.77 (0.72–0.82)
ADA questionnaire (1)	≥6	37.3	65.3	72.7	45.8	85.5	0.73 (0.67–0.78)
Bang et al (2)	≥2	43.9	74.6	67.0	44.4	88.1	0.75 (0.69–0.80)
AUSDRISK (3)	≥13	37.3	72.0	75.1	50.6	88.3	0.80 (0.76–0.85)
FINDRISC (4)	≥5	45.2	75.4	65.5	43.6	88.3	0.78 (0.73–0.83)
Danish Risk Score (5)	≥18	39.7	71.2	71.5	46.9	87.5	0.75 (0.70–0.81)
DESIR (6)	≥3	41.7	63.6	66.1	39.9	83.7	0.71 (0.66–0.77)
Rotterdam Prediction Model 1 (7)	≥6	12.2	25.4	92.5	54.5	77.8	0.56 (0.50–0.63)
**Metabolic syndrome^k^**							
IDRS (14)	≥60	49.0	81.8	65.3	50.7	89.1	0.83 (0.79–0.87)
Ramachandran et al (15)	≥22	48.6	78.8	64.6	49.3	87.5	0.79 (0.75–0.84)
Chaturvedi et al (16)	≥21	34.6	75.9	83.4	66.7	88.8	0.87 (0.84–0.91)
Aekplakorn et al (17)	≥7	45.5	81.0	70.1	54.1	89.4	0.83 (0.79–0.87)
ADA questionnaire (1)	≥6	37.3	56.2	71.0	45.8	78.8	0.65 (0.59–0.71)
Bang et al (2)	≥2	43.9	63.8	64.9	45.4	79.7	0.65 (0.59–0.71)
AUSDRISK (3)	≥11	48.1	81.8	66.6	51.6	89.3	0.82 (0.78–0.86)
FINDRISC (4)	≥4	55.4	94.2	61.5	51.6	96.0	0.84 (0.80–0.88)
Danish risk score (5)	≥13	55.0	77.4	54.8	42.7	84.7	0.70 (0.64–0.75)
DESIR (6)	≥3	41.7	89.1	79.0	64.9	94.3	0.91 (0.89–0.94)
Rotterdam Prediction Model 1 (7)	≥6	12.2	13.9	88.5	34.5	70.2	0.41 (0.35–0.46)

Abbreviations: PPV, positive predictive value; NPV, negative predictive value; AROC, area under the receiver operating characteristic curve; CI, confidence interval; IDRS, Indian Diabetes Risk Score; ADA, American Diabetes Association; AUSDRISK, Australian type 2 Diabetes Risk Assessment Tool; FINDRISC, Finnish Diabetes Risk Score; DESIR, Data From the Epidemiological Study on the Insulin Resistance Syndrome; HDL, high-density lipoprotein.

a Score value with maximum sensitivity and specificity.

b Proportion of people requiring confirmatory biochemical testing.

c True positives/true positives + false negatives.

d True negatives/true negatives + false positives.

e True positives/true positives + false positives.

f True negatives/true negatives + false negatives.

g Derived by plotting 1-specificity on the *x*-axis and sensitivity on the *y*-axis.

h Fasting plasma glucose ≥126 mg/dL or on antidiabetes medication, or both.

i Clinical risk score.

j Impaired fasting glucose (fasting plasma glucose 110–125 mg/dL and not on antidiabetes medication) or diabetes (fasting plasma glucose ≥126 mg/dL or on antidiabetes medication, or both).

k Three or more of the following: raised triglycerides (≥150 mg/dL or treatment of this lipid abnormality), reduced HDL cholesterol (<40 mg/dL in men and <50 mg/dL in women, or treatment of this lipid abnormality), raised blood pressure (systolic blood pressure ≥130 mm Hg or diastolic blood pressure ≥85mm Hg or treatment of previously diagnosed hypertension), or raised fasting plasma glucose (≥100 mg/dL or previously diagnosed type 2 diabetes).

**Table 2:** Range of AROCs of Asian and White DRSs for Diabetes, Dysglycemia, and Metabolic Syndrome Among 451 Participants in Rural Kerala, India

Outcome Variable	Range of AROCs of Asian DRSs	Range of AROCs of White DRSs^a^
Diabetes^b^	0.776–0.802	0.716–0.828
Dysglycemia^c^	0.771–0.801	0.713–0.804
Metabolic syndrome^d^	0.793–0.874	0.651–0.911

Abbreviations: AROCs, areas under the receiver operating characteristic curves; DRSs, diabetes risk scores.

a Excluding Rotterdam Prediction Model 1 (7).

b Fasting plasma glucose ≥126 mg/dL or on antidiabetes medication, or both.

c Impaired fasting glucose (fasting plasma glucose 110–125 mg/dL and not on antidiabetes medication) or diabetes (fasting plasma glucose ≥126 mg/dL, or on antidiabetes medication, or both).

d Three or more of the following: raised triglycerides (≥150 mg/dL, or treatment of this lipid abnormality), reduced HDL cholesterol (<40 mg/dL in men and <50 mg/dL in women, or treatment of this lipid abnormality), raised blood pressure (systolic blood pressure ≥130 mm Hg or diastolic blood pressure ≥85 mm Hg or treatment of previously diagnosed hypertension), or raised fasting plasma glucose (≥100 mg/dL or previously diagnosed type 2 diabetes).

## Discussion

Our study compared the screening performance of Asian and white DRSs for diabetes, dysglycemia, and metabolic syndrome in rural India and found them similar. They were similar because most variables in Asian and white DRSs were associated with all outcome measures and because white DRSs shared all variables of Asian DRSs. This finding is in agreement with a study from Taiwan that showed that DRSs developed in different populations could perform well in detecting diabetes, metabolic syndrome, and chronic kidney disease (8). Conversely, a risk score developed in whites did not perform well in other populations of diverse racial/ethnic origins because of variation in distribution of risk factors and their effect on diabetes in racial/ethnic groups (9). Future research is required to examine whether modifying white DRSs according to the characteristics of Asian populations could enhance their screening performance.

Our study has limitations. For the American Diabetes Association questionnaire we did not have data on macrosomic infant to include in scoring. For the Finnish Diabetes Risk Score (FINDRISC), we did not have data on ever having used antihypertensive medication, and we replaced these with data on current use for scoring. We used the FINDRISC definition of physical inactivity in DRSs that had physical activity as a component, which may have resulted in misclassification.

In conclusion, Asian and white DRSs performed similarly in detecting diabetes, dysglycemia, and metabolic syndrome in rural India.
