# Erratum, Vol. 10, March 21 Release

**DOI:** 10.5888/pcd10.120131e

**Published:** 2013-07-11

In the article “Screening Performance of Diabetes Risk Scores Among Asians and Whites in Rural Kerala, India,” an omission occurred in the Acknowledgments section. That section should read:

“The research had no external funding. We thank all the study participants. Thirunavukkarasu Sathish is supported by the ASCEND Program funded by the Fogarty International Center, National Institutes of Health, under award no. D43TW008332. This article was presented as a poster display in the World Diabetes Congress organized by the International Diabetes Federation December 4–8, 2011, in Dubai, United Arab Emirates.”

The correction was made to our website on July 11, 2013, and appears online at http://www.cdc.gov/pcd/issues/2013/12_0131.htm. We regret any confusion or inconvenience this error may have caused.

